# On the Misrepresentation of Mr. Walker, with Some Remarks on the Defence of His Variolous Essence

**Published:** 1815-02

**Authors:** James Woodham

**Affiliations:** West Smithfield


### *For the Medical and Physical Journal.*

### *On the Misrepresentation of Mr. Walker, with some Remarks on the Defence of his Variolous Essence;* by Mr. Woodham.

"Errors in science have almost universally been the offspring of false or imperfect analogies."

IT is the usage of the two Houses of Parliament, to allow no member to speak twice, excepting the proposer of the question, unless by way of explanation or correction. The same custom, I think, should regulate the writers on disputed points. No one but the author of the subject controverted should be permitted to reply, except where his opponents have been misunderstood or misrepresented. Standing in this last predicament, I trust I shall be granted the privilege of correcting that misrepresentation, and at the same time to make a few remarks on Mr. Walker's defence of his *variolous essence*.

This gentleman, (I shall take leave to employ his own phraseology,) by way of introduction to his observations on vaccination, in the last Number of the Journal, after giving the title of my communication, says, "Had this gentleman passed over this hypothesis, as he considers it, he would hereafter have found, that I am as adverse to any preconceived theories or hypotheses as himself or any other person can be."

How I, or any one, save and except this gentleman himself, could otherwise consider it, I am at a loss to imagine; and, whether it were *præ-conceived*, or *post-conceived*, it is still the same. It is true, he may, if he please, call his *essence* an established fact, but, till I have some better evidence than mere assertion, I must be permitted to regard it as a pure hypothesis.

He continues, "with respect to the quotations this gentleman has made respecting my opinions in this instance, and his remarks upon them, I shall pass over them all, one excepted, being well aware that such opinions of mine as are there given, should they be ill-founded, cannot be productive of any evil tendency in practice." It is impossible to say that they are either ill or well-founded, because we have no means of determining; but that they cannot be productive of practical evil, is somewhat problematical. The hypotheses of this gentleman, may, like those of his hypothetical prototype Sydenham, sit so loosely about him, that he can throw them off when they will not bend to experience, or not suffer them to influence his practice; but it is far from

 impossible, impossible, that some young men of confined education, and practising in remote villages, may, on perusing his paper, become so captivated with his *essential* hypothesis, that, like the adopters of Sydenham's, they may wish to make their practice and that accord; and, instead of cooling their variolous patients both within and from without, they may place them in such a degree of heat, and administer such calorific medicines, as they suppose will promote the fermentation of the *essence*, and its expulsion from the system. This, however improbable, is, as I before mentioned, not impossible, and, should only *one* fatal instance of such treatment reach this gentleman's ears, "sage grave man" as I doubt not he is, what will be the reproaches of his moral sense, and how much will he lament that he had ever sported in the fields of hypothesis!

"The circumstance alone," he goes on to observe, "which I shall notice, is the following observation, produced in the manner of analogy, thus: 'As well might it be said that this essence or innate principle is an alkali, and that the exciting cause is an acid, that coming in contact, an effervescence or commotion takes place in the lymph or blood, and a neutral salt is formed, which, being expelled from the system, leaves the patient ever after secure from the disease.'"

This is the misrepresentation of which I have to complain, and to correct which I have resumed my pen. It is asserting that to have been used as an analogy, which must have struck every one was employed as a ridiculous hypothesis, to show that it was equally rational and probable, or rather, though grossly absurd and improbable, not more absurd and improbable than his own.

Rejecting, therefore, what he has *mis-named* my analogy, this gentleman thus introduces the following, of which, he is pleased to say, he is indebted to me for the hint. "Without remarking upon this supposed analogy, which is by no means congenial with my ideas of analogies, were I to attempt to elucidate my opinion respecting a prior existence in the system of the essence, or an innate principle of small-pox, its excitement into action, and its expulsion, by a chemical analogy, I should adduce the example of what happens in the instance of fermenting liquors, as beer, &c. The essence or innate principle of fermenting matter, as we well know, exists naturally in the beer; but, in order to expel or drive out this essence or innate principle, remaining dormant or latent, and from which it is necessary it should be purified by art, a small portion of the same essence, in the form of yeast, which had been previously produced by a similar process, is added—the desired ferment or commotion is excited, the original principle of fermentation existing in the beer is collected together, and finally expelled, or driven out, and used in a future similar purpose, and so on *ad infinitum*."*

The above, with the note below, may be not inaptly termed, I think, this gentleman's *congenial* analogy and illustration, and which he considers, I presume, as not only correct but complete: "*totum in toto, et totum in qualibet parte.*" Let us, therefore, examine this analogy a little; let us bring it to its proper test, and see whether it is founded, as a correct analogy should be, on a *real*, not a *nominal*, resemblance. Is there any *real* resemblance, then, I would ask, between a beer vessel, which consists of a few staves hooped together with iron or wood, and having a top and bottom, and the "human form divine," composed of its bones, muscles, &c.?—Is there any *real* resemblance between the beer this vessel may contain, and the blood and other fluids of the body?—Is there the smallest *real* similitude between a patient in the pustular stage of small-pox, and a barrel of ale or porter in a state of fermentation, with the yeast running out at an aperture vulgarly called a bung-hole?—Who of unclouded intellect, who that has not suffered his imagination to outstrip his reason, will answer in the affirmative?—If, then, there be only a *nominal* resemblance, what becomes of this gentleman's *congenial* analogy, so triumphantly brought forward in support of his *essence*?—What becomes of the carbo-oxygenated solution of my "supposed paradox or inconsistency?"—Why, like the weird sisters, vanished into air; for

> "The mind hath bubbles, as the water has,
> And these are of them:——."

*West Smithfield,* JAMES WOODHAM.

*Jan.* 6, 1815.

---

* "I have taken *malt liquor* as an example, ———. The essence or innate principle in this instance I consider to be existing originally in the wort, viz. carbon and oxygen, the union of which, forming carbonic acid, is the natural or inherent cause of fermentation, which produces and throws out what is called yeast; and which is commonly used as a ferment, in the purpose of promoting fermentation in future processes of the same nature. Here, then, we have, by analogy, I think, a solution of this gentleman's supposed paradox or inconsistency, viz. 'It is, therefore, according to circumstances, both agent and patient, cause and effect, the excitement to action, and the recipient of that excitement:' and this I take to be the case likewise in inoculated small-pox, and in vaccination."

